# A Hierarchical Bayesian Model to Quantify Uncertainty of Stream Water Temperature Forecasts

**DOI:** 10.1371/journal.pone.0115659

**Published:** 2014-12-26

**Authors:** Guillaume Bal, Etienne Rivot, Jean-Luc Baglinière, Jonathan White, Etienne Prévost

**Affiliations:** 1 INRA, UMR 0985 ESE Ecologie et Santé des Ecosystèmes, Rennes, France; 2 Marine Institute, Oranmore, Ireland; 3 Agrocampus Ouest, UMR 0985 ESE Ecologie et Santé des Ecosystèmes, Rennes, France; 4 INRA, UMR 1224 Ecobiop Ecologie Comportementale et Biologie des Populations de Poissons, Saint Pée sur Nivelle, France; 5 Université de Pau et des Pays de l′Adour, UMR 1224 Ecobiop Ecologie Comportementale et Biologie des Populations de Poissons, Anglet, France; Technical University of Denmark, Denmark

## Abstract

Providing generic and cost effective modelling approaches to reconstruct and forecast freshwater temperature using predictors as air temperature and water discharge is a prerequisite to understanding ecological processes underlying the impact of water temperature and of global warming on continental aquatic ecosystems. Using air temperature as a simple linear predictor of water temperature can lead to significant bias in forecasts as it does not disentangle seasonality and long term trends in the signal. Here, we develop an alternative approach based on hierarchical Bayesian statistical time series modelling of water temperature, air temperature and water discharge using seasonal sinusoidal periodic signals and time varying means and amplitudes. Fitting and forecasting performances of this approach are compared with that of simple linear regression between water and air temperatures using i) an emotive simulated example, ii) application to three French coastal streams with contrasting bio-geographical conditions and sizes. The time series modelling approach better fit data and does not exhibit forecasting bias in long term trends contrary to the linear regression. This new model also allows for more accurate forecasts of water temperature than linear regression together with a fair assessment of the uncertainty around forecasting. Warming of water temperature forecast by our hierarchical Bayesian model was slower and more uncertain than that expected with the classical regression approach. These new forecasts are in a form that is readily usable in further ecological analyses and will allow weighting of outcomes from different scenarios to manage climate change impacts on freshwater wildlife.

## Introduction

Climate change [1], [2] is impacting the physiology, phenology and distributions of organisms worldwide resulting in changing communities structure [3], [4]; stream ecosystems are no exception [5] with climatic changes affecting both water temperatures and discharge, key factors in the functioning of freshwater ecosystems [6], [7], [8]. In particular, water temperature is of primary importance for ectothermic organisms having limited ability to adapt their spatial distributions owing their dependence on river networks and habitat fragmentation [9]. For instance, changes in water temperature affects the growth of cold water fish such as salmonids [10], [11], [12] and may disrupt their life histories and population dynamics [13], [14], [15]. Such changes may result in modifications of the distributions [16], [17], [18] and ranges [19], [20], [21] of native species, while invasive species could be favoured [22], [23], [24]. This in turn can alter the structure and functioning of ecosystems, food web architecture, dynamics and energy budgets [24], [25].

Long and continuous historical water temperature time series are required to assess how fluctuations of water temperature have affected the functioning of aquatic ecosystems, while forecast scenarios are prerequisite to evaluate possible impacts of future global warming. Freshwater water temperature time series are however, comparatively rare, shorter, and more prone to errors than time series of air temperature. In addition stream water temperature is rarely available as an output of climate change models. Providing tools to reconstruct and generate time series scenarios of water temperature based on commonly available predictors, such as air temperature and/or water discharge [26], [27], [28], is thus a key issue.

Both mechanistic and statistical modelling approaches have been developed to predict water temperature [7]. Mechanistic models based on energy budgets are data-intensive, requiring site-specific and often costly data such as meteorological variables other than air temperature, topography and stream bed information [29], [30], [31], [32]. They provide fine scale estimates of the water temperature but are difficult to use for forecasting stream temperatures on wider geographical scales because of the amount of data needed.

Statistical models are often deemed more robust, requiring air temperature and in some instances water discharge data, and tend to be more popular for water resource and aquatic habitat management [7], [33]. After filtering long term trends, time series models consider random variations in water temperature as a function of random variations in air temperature [7], [34], [35], [36]. These methods perform well in filling gaps in past series of water temperature when continuous series of air temperature are available, but are inadequate in forecasting water temperature over periods of several years. Periodic autoregressive models [37] and non-parametric models (e.g., based on k-nearest neighbours methods) and artificial neural networks [38] have also been proposed but they suffer from a lack of parsimony and may provide spurious predictions outside the range of temperatures used for model fitting [33].

Simple linear regression models between air and water temperatures have been one of the most used approaches to infer water temperature [39], [40], [41], [42], [43]. Because of their simplicity and low data requirement, these simple approaches are quite popular among freshwater ecologist to reconstruct and/or forecast stream temperature time series[44], [45], [46]. However, simple regression models suffer from methodological caveats that have received little attention in the literature. Both air and water temperature signals show seasonal fluctuations with maximum amplitudes of approximately 15 to 25°C. The synchrony between the two signals due to seasonal fluctuations will thus result in strong positive correlations between pairwise records of water and air temperatures that could hide joint patterns of the evolution of air and water temperatures over longer time scales [34], [35], [40], [47]. Simple models based on this positive correlation could thus lead to biased water temperature forecasts or to underestimation of the associated uncertainty.

In this paper, we develop a generic statistical approach to forecast water temperature from air temperature and water discharge time series that allows the components of the correlation owing to seasonality to be separated from those owing to longer term fluctuations or trends. The approach is developed in a fully hierarchical Bayesian framework that provides a probabilistic rationale to propagate and quantify uncertainty around inferences and predictions [48]. To demonstrate the potential of our approach we compared its performance to that of simple linear regression over a short time scale on i) a simulated example, and ii) time series of air temperatures, water discharge and water temperatures from three French coastal streams of contrasting size and bio-geography.

## Material & Methods

### 1. Modelling and forecasting water temperature

#### Limits of simple regression models: a simulated illustrative example

**Figure 1:**
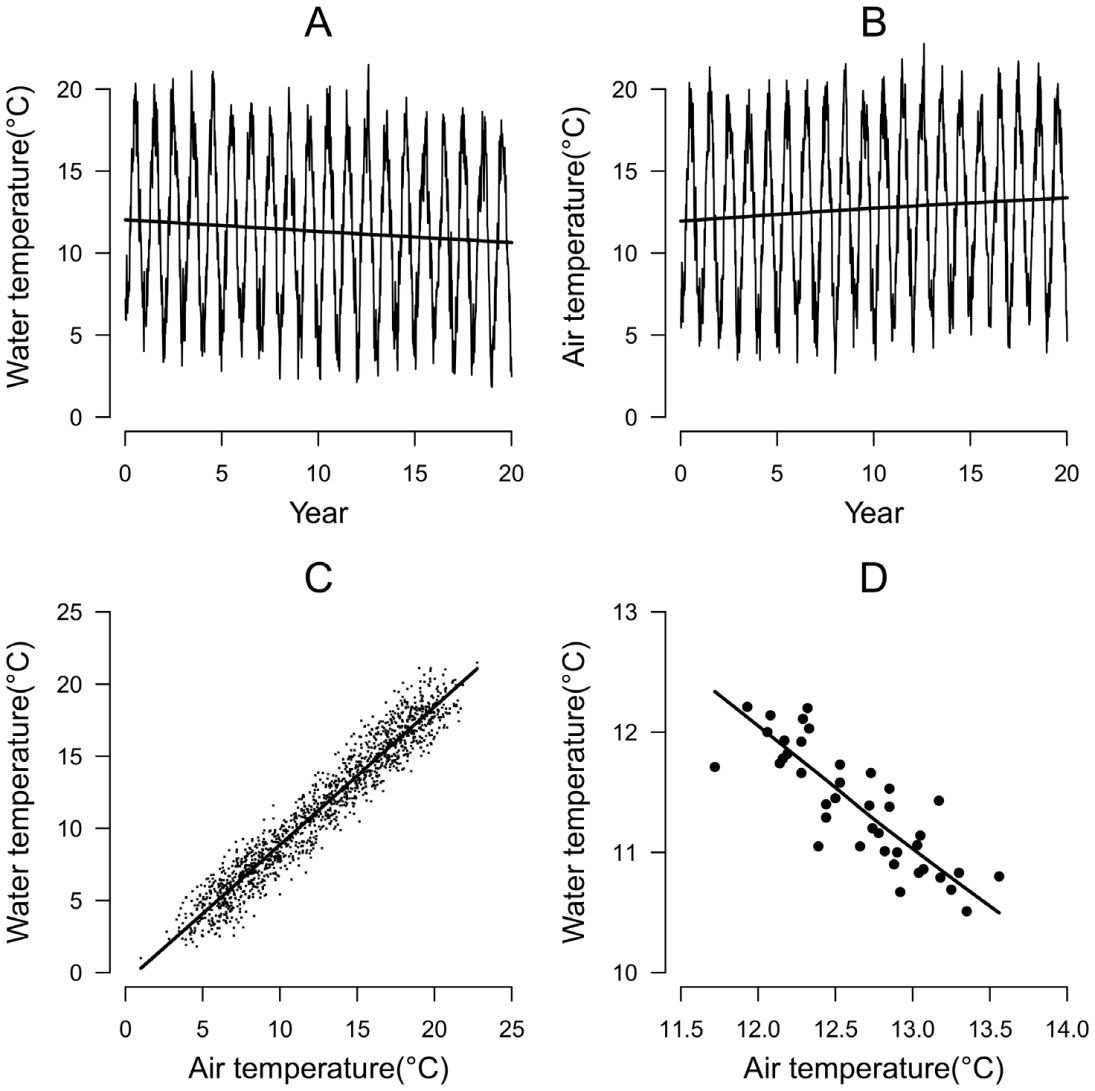
Linear regressions between pairwise records of air temperatures and water temperatures at small time scale (moving average over 5 days; panel C) and long time scale (moving average over 6 months; panel D).

To illustrate the potential caveats of simple regression models between water and air temperature, we simulated water and air temperature time series exhibiting characteristic seasonal fluctuations and weak but opposite long term time trends (Fig. 1). Daily water temperatures were simulated using a sinusoidal function with an annual periodicity and amplitude of 13°C, plus a negative trend in the annual mean of −0.5°C over 20 years starting from an original mean of 12°C. The time series of air temperature was simulated with the same periodicity and amplitude but with an increasing trend of +0.5°C over the 20 years. Both time series were augmented with noise modelled as a first order autoregressive model with autocorrelation fixed to 0.5 and variance of the innovation process fixed to 2. Because seasonal fluctuations are synchronous and exhibit a much higher amplitude (13°C) than the change in annual means, pairwise correlation between air and water temperatures considered over a short time period (5 day moving average in this example) is positive (r = 0.97, *p*<0.001), masking the negative correlation, which was weaker, but still significant (r = −0.81, *p*<0.001) between the moving average temperatures calculated over 6 months periods.

This simulated example illustrates that relying on only the strength of the short term correlation to forecast water temperature could be inappropriate as it may lead to erroneous conclusions regarding the long term evolution of water temperature. By contrast, correlations calculated over a longer time period will only pick up the associations between signals with longer periodicity. Neither of these approaches however, allows both the seasonal and longer term trends to be simultaneously captured. Moreover, simple regressions between air and water temperatures do not account for the influences of other covariates such as water discharge. A more consistent statistical approach should account for both seasonality and long term trends in the climatic time series and the potential effect of water discharge as a covariate.

#### Separating seasonality from longer time trends: model M_1_

Model M_1_ is a fully Bayesian hierarchical model based on time series decomposition according to eq (1) and is composed of three modules which are all embedded within the same hierarchical model (detailed hereafter). The first module aims at decomposing the time series of predictors (air temperature and water discharge) and response variable (water temperature) into long term trends, seasonal fluctuations and residual variability; In the second module, some relations are introduced in the hierarchical structure to characterize the relationships between the time series of the water temperature and its predictors at different time periodicity; In the third module, these relationships are used to forecast water temperature from air temperature and water discharge.

In module (1), air temperature and log-transformed river flow time series were decomposed using eq. (1) [34], where *X_y,t_* represents the time series of a variable (indifferently air temperature, water temperature or (log-transformed) water discharge).
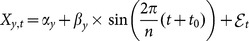
(1)


*α_y_* and *β_y_* are the mean and amplitude on the time window *y* respectively. A five-day arithmetic average was used as the short time step *t* so n is equal to 73. A long-term window of 6 months was set for the longer time window *y.* Preliminary trials have shown that averaging over five days was the best compromise between the computational performances (the computational time increases with the number of time steps) and the degree of smoothing of short term variability. Using long term windows of 6 months (instead of 1 year) maximizes the number of time step used to fit relationships in eqs. (2a)-(2b) (see hereafter). 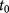 sets the position of the sine signal on the time line. Preliminary analyses using parameters 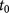 that vary between periods *y* have shown only very slight variability of 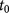 between periods (not shown), and 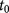 was therefore assumed constant in time. As residual random terms of the three time series are known to exhibit significant positive autocorrelation, random terms 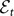 were modelled as a first order autoregressive process with autocorrelation coefficient 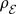 and variance of the innovations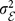. Time series of random terms were modelled as mutually independent between the time series (water and air temperature, water discharge). Measurement errors were not included in the model. As observation errors were quite low when compared to the natural variability on our three case study (see Annexe 1), they were unlikely to impact on the results and have not been modelled. Priors specified for the unknown parameters 
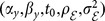
 were all weakly informative (Table 1).

**Table 1:** Prior distributions assigned on parameters in models *M_0_* and *M1*.

Model	Time series	Parameter	Prior
*M_0_*	*_WT_*	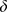	∼ Normal (E = 0, VAR = 1000)
*M_0_*	*_WT_*	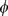	∼ Normal (E = 0, VAR = 1000)
*M_0_*	*WT*	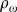	∼ Uniform [−1, 1]
*M_0_*	*WT*	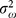	∼ Gamma (E = 1, C.V. = 1000%)
*M_1_*	*AT*	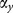	∼ Normal (E = 0, VAR = 1000)
*M_1_*	*AT*	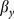	∼ Uniform[0, 20]
*M_1_*	*AT*	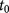	∼ Uniform[45], [65]
*M_1_*	*AT*	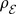	∼ Uniform [−1,1]
*M_1_*	*Q*	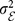	∼ Gamma (E = 1, C.V. = 1000%)
*M_1_*	*Q*	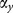	∼ Normal (E = 0, VAR = 1000)
*M_1_*	*Q*	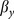	∼ Uniform[0, 20]
*M_1_*	*Q*	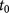	∼ Uniform[15], [25]
*M_1_*	*Q*	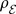	∼ Uniform [−1, 1]
*M_1_*	*Q*	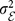	∼ Gamma (E = 1, C.V. = 1000%)
*M_1_*	*WT*	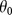	∼ Normal (E = 0, VAR = 1000)
*M_1_*	*WT*	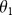	∼ Normal (E = 0, VAR = 1000)
*M_1_*	*WT*	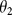	∼ Normal (E = 0, VAR = 1000)
*M_1_*	*WT*	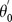	∼ Normal (E = 0, VAR = 1000)
*M_1_*	*WT*	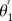	∼ Normal (E = 0, VAR = 1000)
*M_1_*	*WT*	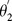	∼ Normal (E = 0, VAR = 1000)
*M_1_*	*WT*	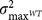	∼ Gamma (E = 1, C.V. = 1000%)
*M_1_*	*WT*	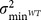	∼ Gamma (E = 1, C.V. = 1000%)
*M_1_*	*WT*	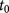	∼ Uniform[45], [65]
*M_1_*	*WT*	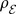	∼ Uniform[−1, 1]
*M_1_*	*WT*	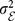	∼ Gamma (E = 1, C.V. = 1000%)

E, VAR and C.V/correspond to expectation, variance and coefficient of variation respectively.

In module (2) of the hierarchical model *M_1_*, parameters describing the shape of the sine function for the water temperature were modelled a priori as linear functions of those describing the shapes of the sine functions of air temperature and water discharge. The sine signal can be parameterized by any combination of two of these four parameters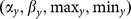, where 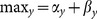 and 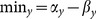 are the maximum and the minimum of the signal, respectively. Here, the sine signal of water temperature (WT) was parameterized in terms of maximum 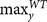 and minimum 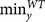 allowing the impact of air temperature (AT) and discharge (Q) to be integrated. 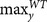 was defined as a linear function of the maximum air temperature and minimum water discharge (eq. (2a)), while 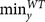 was defined as a linear function of the minimum air temperature and maximum water discharge (eq. (2b)):
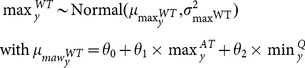
(2a)

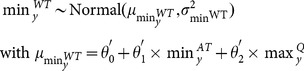
(2b)


Previous analyses have shown that the best compromise between the complexity of the relationships and the quantity of deviance explained was obtained through this set of simple linear regressions. Moreover, eqs (2a)-(2b) are quite interpretable in term of environmental processes. 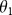 is expected to be positive as 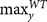 series is expected to be positively correlated with the 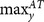 series. 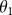 is expected to be negative as high minimum discharge is expected to decrease the seasonal variation of water temperature because higher minimum water discharge generally corresponds to cool temperatures in summer. 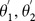 are both expected to be positive, as warmer air relates to warmer water and a high water discharge in winter tends to relate to high water temperatures because high winter flows correlate with wet and mild conditions 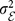. Weakly informative prior distributions were set for unknown parameters 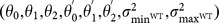 (Table 1). As stated in the above section, random terms in water temperature were modelled as a first order autoregressive process assumed to be independent from that of the air temperature and flow time series'. Weakly informative parameters were set on related parameters (Table 1). Observation errors were not modelled.

Finally module (3) is a forecasting module embedded in the hierarchical model *M_1_* that uses the information from module (1) and (2) to forecast the water temperature while fully propagating the uncertainty through the hierarchical structure. We denote *AT′* and *Q′* the time series' of air temperature and water discharge from which we want to forecast water temperature WT′. Such series may be historical observations or climatic model scenarios. *AT′* and *Q′* are first decomposed following the model described in eq. (1) to obtain estimates of parameters 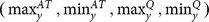. Relationships (2a)-(2b), combined with the regression parameters 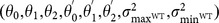 are then used to forecast parameters 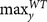 and 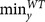
series, which characterise the forecast sine signal of WT′. Combined with the variance of noise around this, the whole WT′ series can be forecast conditionally upon all the information conveyed by the historical series of water temperature, air temperature and water discharge and upon *AT′* and *Q′* series. In a Bayesian framework, this is done through the posterior predictive distribution [49]of the forecast time series WT′, that integrates out the uncertainty around all model parameters (from their posterior distribution) and the uncertainty owing to the autoregressive residual variation. Posterior predictive distributions were also used to directly estimate any missing data.

#### A simple linear regression model

For the purpose of comparison, a linear regression model between water and air temperatures (*M* ***_0_***) was also implemented and fitted in a Bayesian framework using pairwise historical records averaged over 5 days using the following equation:
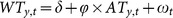
(3)


Residual random terms 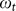 were modelled as a first order autoregressive process with autocorrelation coefficient 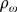
and variance of the innovations 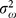. Non informative prior distributions were used for parameters in eq. (3) (Table 1). Posterior distributions of the parameters 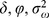 and the series of air temperature *AT′* were used to provide posterior predictive distributions of the forecasted time series WT′.

### 2. Bayesian computation

Bayesian fitting, forecasting and the derivations were implemented using Markov Chain Monte Carlo algorithms in JAGS (Just Another Gibbs Sampler) [50] through the R software [51]. Three parallel MCMC chains were run and 20,000 iterations from each were retained after an initial burn-in of 20,000 iterations. Convergence of chains was assessed using the Brooks-Gelman-Rubin diagnostic [52].

### 3. Posterior checking, model comparison and cross validation

The consistency between the model a posteriori and the data was assessed via posterior checking techniques [49]. If model fits, then replicated data generated under the model should look similar to observed data, i.e. data should look plausible under the posterior predictive distribution. Any systematic discrepancy coming from this “self consistency” check indicates potential failing of the model. Here, we employ as a discrepancy measure the χ^2^ discrepancy statistic [49]. For each model, the χ^2^ statistic was calculated as:
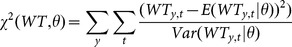
(4)where 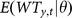 and 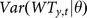 are respectively the expected mean and variance of the water temperature conditionally upon the parameters *θ*. For each set of parameters (ψ) drawn in their joint posterior distributions, the realized discrepancies 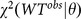 computed with the observed values of water temperature were compared against the predicted discrepancies 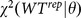 computed with posterior predictive replicates of water temperature. If the model is consistent with the data, 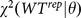 should be similar to 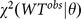. The Bayesian *p*-value was calculated as the probability that 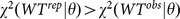 estimated over the posterior sample of *θ.* A *p*-value near 0.5 indicates consistency between model and data, whereas a very high (0.95) or low (0.05) *p*-value provides serious warning.

The Deviance Information Criterion (DIC) [53] was also used to compare the goodness of fit of models *M_0_* and *M_1_*. As with the Akaike Information Criterion, DIC combines a measure of the goodness of fit penalized by a measure of the model complexity. The smaller the DIC, the more a model is supported by data.

The predictive performances were compared through cross-validation analyses. A cross-validation was implemented using the first two thirds of the available time series to fit the model and make forecasting on the last third. These were subsequently compared against known temperatures. Differences between the observed and predicted water temperatures were quantified using the root mean square error (RMSE) [37], [41], [54].

### 4. Application

**Figure 2:**
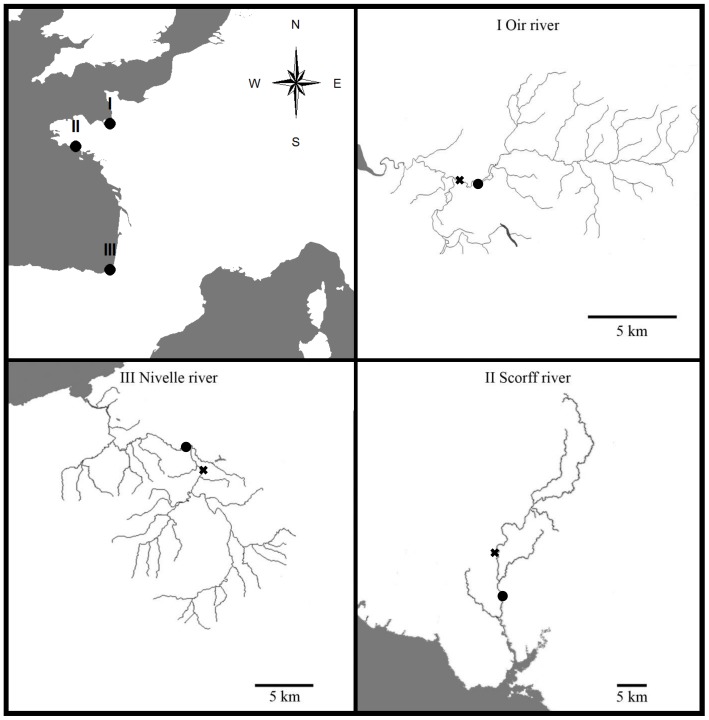
Watersheds of the three case study rivers. x: hydrometric station; •: water temperature measurement stations.

**Figure 3:**
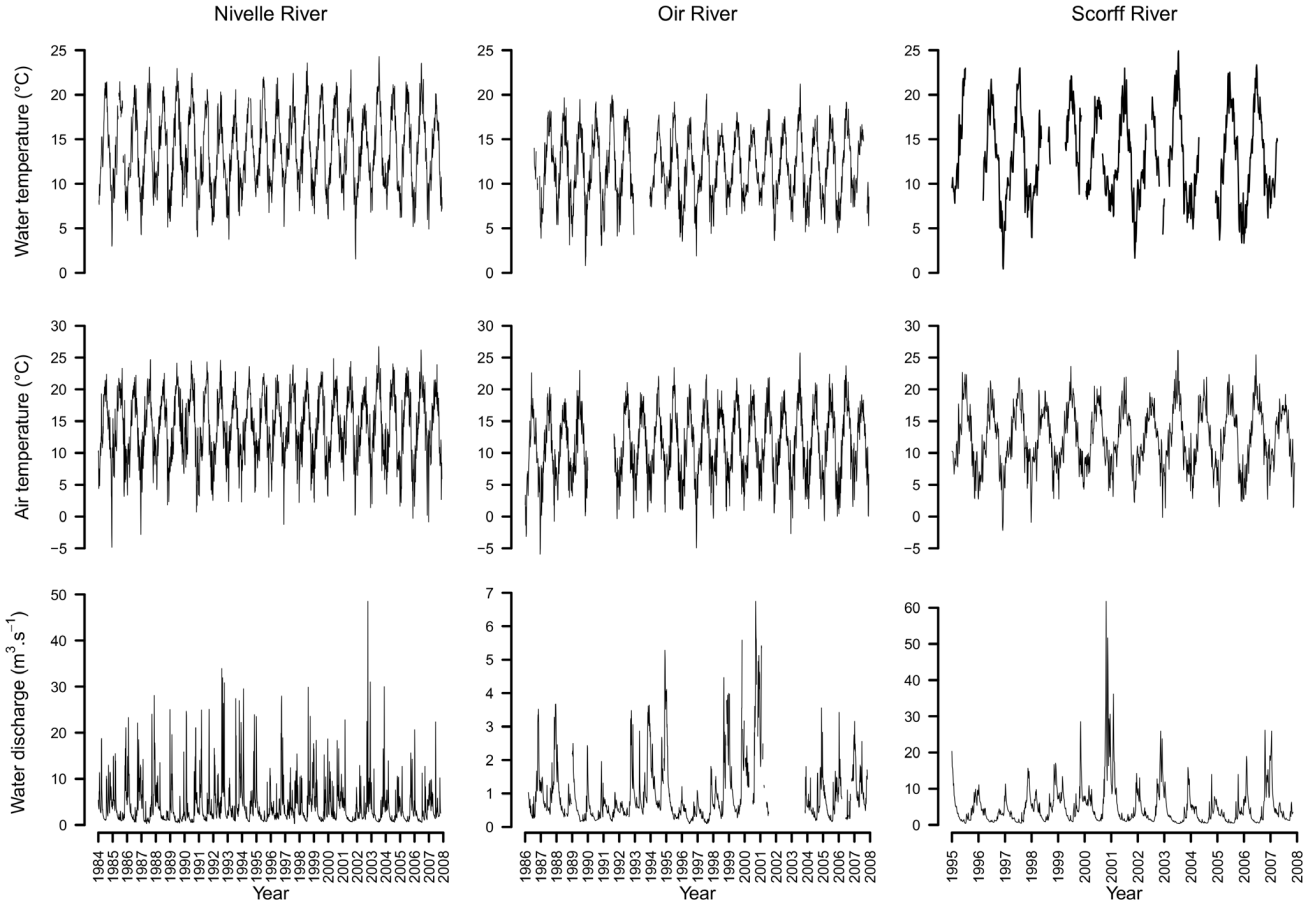
Time series of the available data on the three rivers used as case studies.

Our approach was applied to i) the illustrative simulated time series of air and water temperature and ii) time series of water temperature, air temperature and water discharge from three coastal streams of the French Environmental Research Observatory for Small Coastal Streams (ERO SCT; Fig. 2 which benefits from long term monitoring of environmental parameters and fish populations; the Oir River [55], [56], the Scorff River [57] and the Nivelle River [58]. The main characteristics of rivers and associated data are summarized in Table 2 and Fig. 3. More details about the Rivers are provided in Annexe 1 and data used in this study can be made available by contacting staff members of the ERO SCT (https://www6.inra.fr/ore-pfc).

**Table 2:** Study rivers characteristics and summary of data sets.

River		Oir	Scorff	Nivelle
Location		Lower Normandy	Brittany	Basque Country
Mouth (Latitude & Longitude)		48°37′N, 1°17′W	47°28′N, 3°23′ W	43°22′ N, 1°38 W
Drainage area (km^2^)		87	480	238
River Length (km)		19.5	75	32.2
Estuary length (km)		8	15	8.8
Source altitude (m above sea level)		220	270	600
Geology (predominant)		Schist & granite	Granite & schist	Schist & sandstone
Land use (% of catchment)	Agricultural	82	60	80
	Woodland	10	30	
	Urban	6		
	Wetland	2		
Climate		Oceanic	Mild oceanic	Mild & wet oceanic
Precipitation (mm per year)		∼1000	∼1000	∼1700
Water temperature	Period	1986–2007	1995–2007	1984–2007
	Mean (°C)	11.88	12.88	13.88
	Missing data (%)	9.53	26.98	2
Air temperature	Period	1986–2007	1995–2007	1984–2007
	Mean (°C)	11.24	12.39	14.14
	Missing data (%)	8.03	0.63	0.34
Water discharge	Period	1986–2007	1995–2007	1984–2007
	Mean (m^3^.s^−1^)	0.99	4.95	4.39
	Missing data (%)	17.75	1.05	0.68

### 5. Forecasting scenarios

For the illustrative example based on simulated data, models *M_0_* and *M_1_* were applied to forecast water temperatures, with a simplified version of model *M_1_* (eq.1 to 3) using air temperature as the only predictor of water temperature. Water temperature was forecast based on a 50 years extension of air temperature simulated with a linear increment in the annual mean resulting in a warming of +3.2°C (corresponding to the maximum air temperature increments supported by the IPCC (2007) [1].

The three simulated scenarios, extended from the coastal stream examples, also consisted of 50 years of air and water temperature and discharge data. Air temperatures were simulated with the same +3.2°C linear increment in the annual mean. To allow for comparisons between outputs of models *M_0_* and *M_1_*, the simulated water discharge time series were constructed with a constant mean and amplitude set to the average over the last 10 years.

## Results

First, we compare the forecasts of water temperature provided by the models *M_0_* and *M_1_* with the simple simulated example. Then, we compare the quality of fit, the internal consistencies (posterior checking) and the forecasting performances (cross validation) of the two models when applied on the three coastal streams. Lastly, we provide some key features of the results of the model *M_1_*.

### 1. Simulated illustrative example

**Figure 4:**
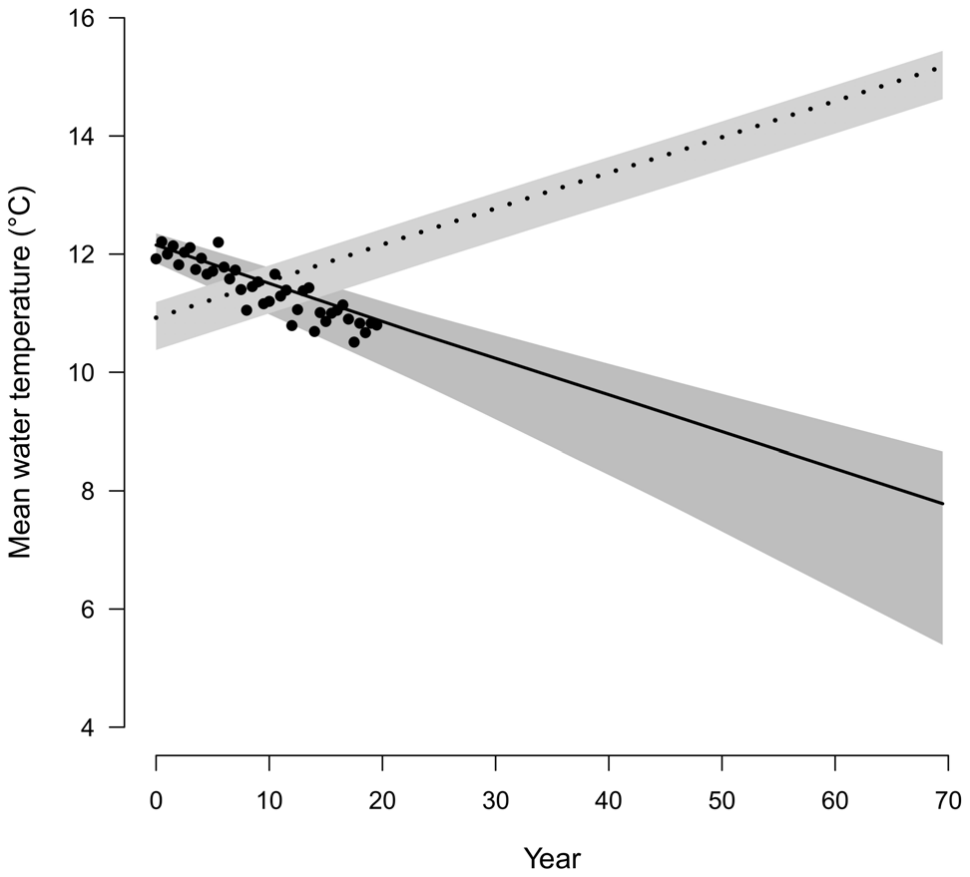
Evolution of temperatures predicted by the modelling approaches *M_0_* and *M_1_* for the simple simulated example. Posterior means are represented by a full line for the model *M_1_* and a dotted line for model *M_0_*. 95% Bayesian credibility intervals corresponds respectively to dark and light grey shaded areas for models *M_1_* and *M_0_*. Points correspond to initial data.

Fig. 4 highlights that the two modelling approaches lead to different forecasts of water temperature. Simulations from *M_0_* are based on the positive correlation between air and water temperatures owing to their synchronous seasonal fluctuations, which logically leads to forecast an increase in the water temperature. By contrast, *M_1_* captures the difference in long term trends in air temperature and water temperature and hence produces a decreasing trend in water temperature, which is more consistent with the observed patterns in the historical time series. The 95% credibility envelope around the forecast produced by *M_1_* is also wider than that produced by *M_0_*.

### 2. Applications to coastal streams data

#### Posterior checking, model comparison and cross validation

Overall, when applied to the data from the three coastal streams, the model *M_1_* produces similar results to *M_0_* in term of internal consistency and outperformed it in terms quality of fit and predictive performance.

**Figure 5:**
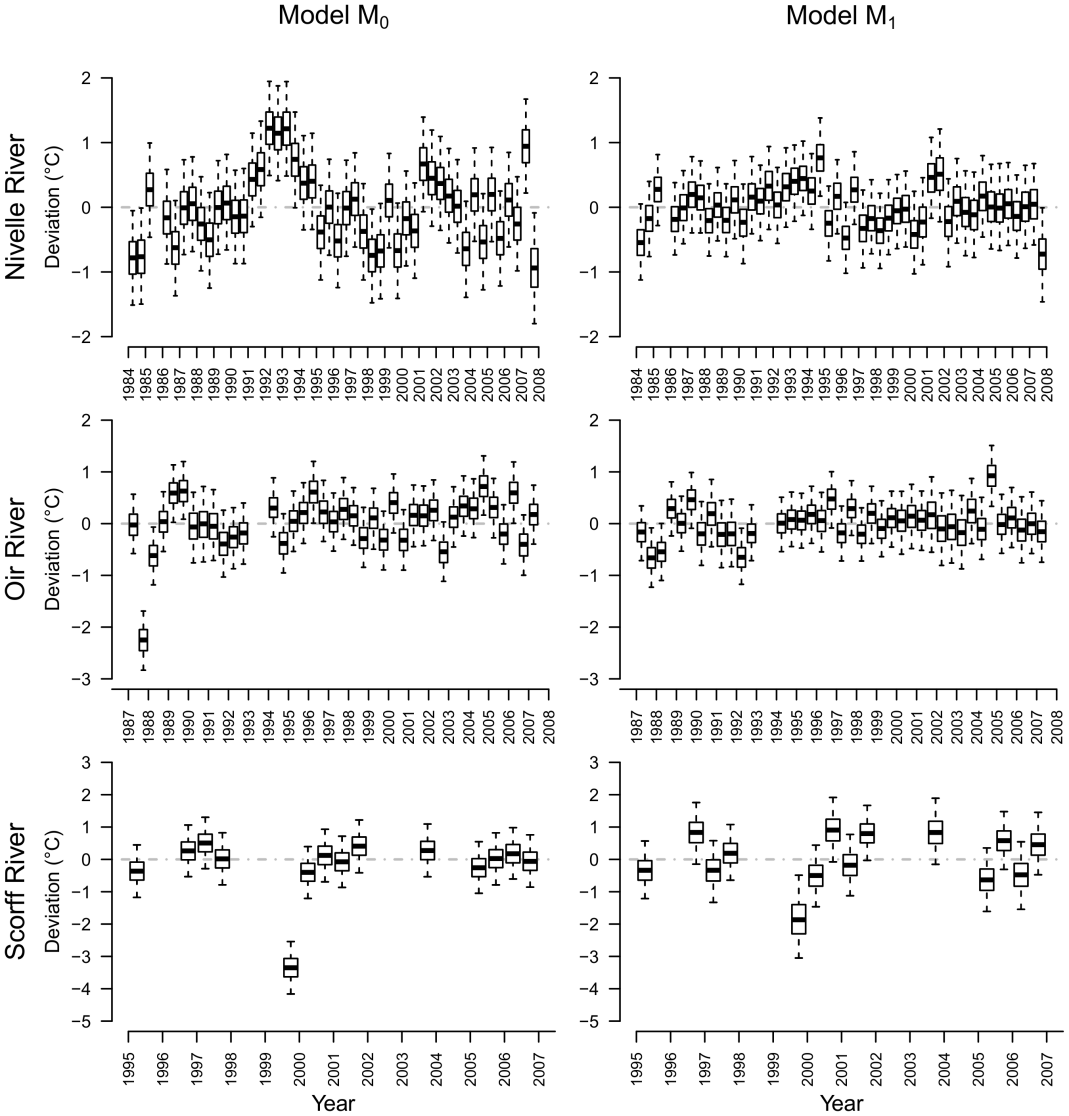
Boxplots of the differences between observed and fitted means of water temperatures by six month blocks on the three Rivers with the modelling approaches *M_0_* and *M_1_*. Only blocks with representative data were included.


For each of the three coastal streams, discrepancies between observed and fitted means calculated on 6 months intervals are globally smaller for model *M_1_* than for model *M_0_* (Fig. 5). This value appeared close to 0.5 in case of both models indicating equivalent posterior consistency with data. It is worth noting that the simple regression approach *M_0_* was not able to capture the variability in the data for the years 1991, 1992 and 1993 for the Nivelle River, 1987 for the Oir River and 1999 for the Scorff River.

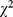
 discrepancies and associated *p*-values for the two models do not reveal inconsistencies between the models and the data from the three rivers as all *p*-values are close to 0.5 (Table 3).

**Table 3:** Model selection, posterior checking and predictive performance for the two modelling approaches applied to the 3 rivers.

River	Model	Dev	pD	DIC	*p*-value	RMSE
**Nivelle**	*M_0_*	12715	6.2	12721	0.50	3.15
	*M_1_*	9882	767	9959	0.50	2.29
**Oir**	*M_0_*	9379	71.8	9451	0.51	2.48
	*M_1_*	7938	68.2	8007	0.49	2.17
**Scorff**	*M_0_*	5200	6.4	5207	0.51	3.40
	*M_1_*	4038	51.9	4085	0.50	3.00

Dev: deviance posterior mean; pD: measure of the model complexity (estimated number of parameters); DIC: Deviance Information Criterion. p-value: p-value for the posterior checking tests; RMSE: root mean square errors used to quantify the predictive performance.

Deviance Information Criterion clearly indicates a better fit of model *M_1_* for the three coastal streams (Table 3). The posterior means of deviances are much greater for model *M_0_*, and differences are not counterbalanced by the higher model complexity of model *M_1_*. The high number of parameters estimated on the Oir River for model *M_0_* (pD = 71.8, Table 3) differs to that of the Scorff and Nivelle Rivers (pD close to 6 in each case, Table 3) and is attributed to the higher frequency of missing air and water temperature data for the Oir River (in the Bayesian framework missing data are considered as unknown values to be estimated).

The RMSE (Table 3), used as a summary measure to quantify the predictive performances of both models, indicates better predictive performances for model *M_1_* on the three rivers. Even when a large proportion of missing data for any one, or for multiple time series' could have impeded the precision in the parameter estimates in eqs. (2a)-(2b), the performance of the new method was superior in its estimates than those of model *M_0._* See for instance air and water temperature data in 1990, 1991, 1992 and 1994 on the Oir River together with the high proportion of missing data for water discharge between 2001 and 2004, which represented nearly half of the forecasting period.

#### Means and amplitudes of the air temperature, water discharge and water temperature time series

**Figure 6:**
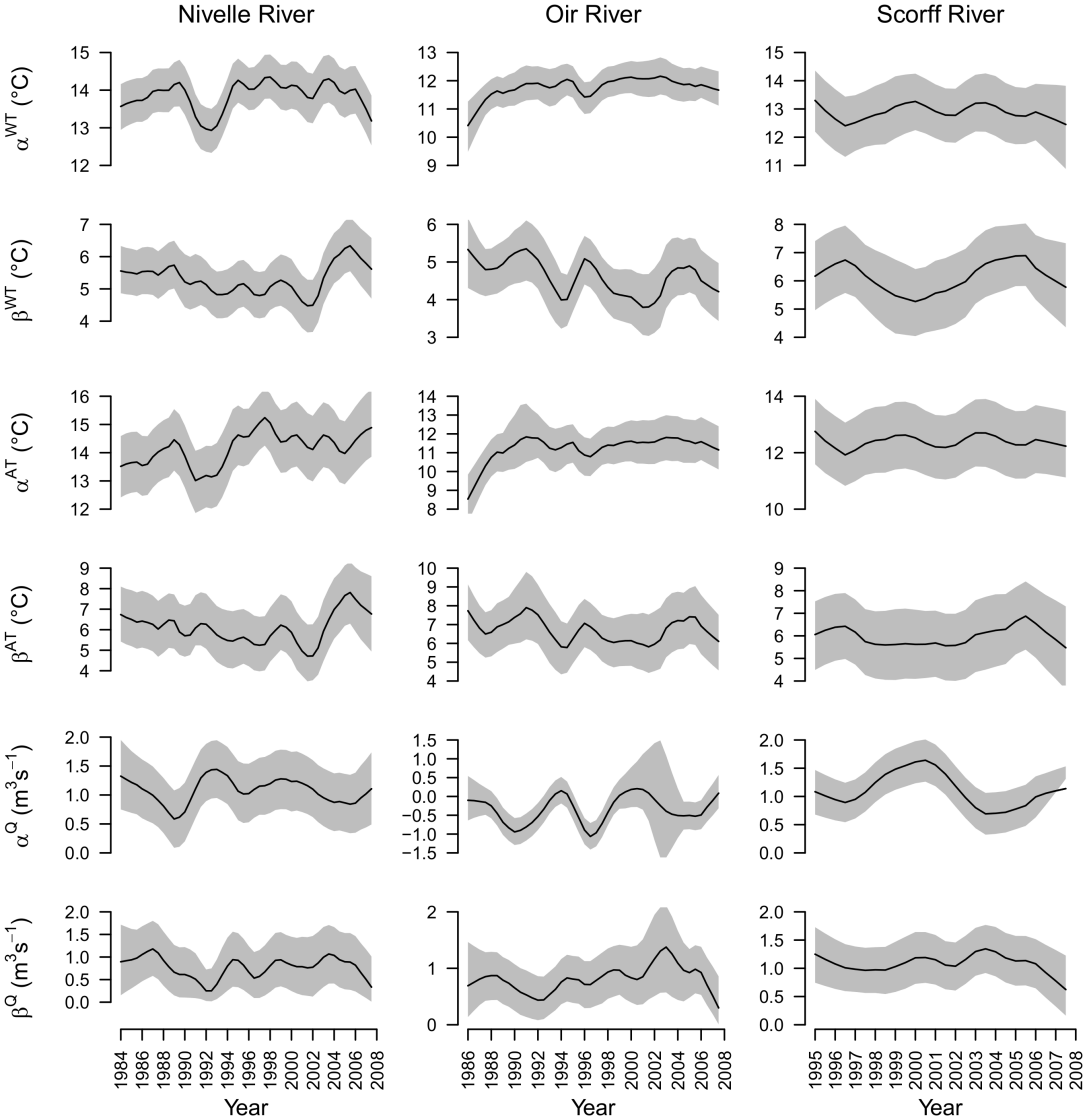
Posterior distribution of the means (*α*) and amplitudes (*β*) characterizing the time series of water temperature (WT), air temperature (AT) and water discharge (Q) on the three rivers. Solid line: posterior medians; shaded area: 95% posterior interval.

Bayesian estimates of means and amplitudes (estimated for every 6 months intervals) vary between time intervals, and with the exception of increasing trends in the mean water temperature of the Oir River and air temperature of the Nivelle and Oir rivers, no other clear trend emerged. Uncertainty around estimates is quite low (Fig. 6) and slightly lower for periods with higher proportion of missing data (see for instance the period 2001–2004 for water discharge on the Oir River).

Overall mean water temperature increased from north (Oir R.) to south (Nivelle R.). Amplitudes of air temperature were close to 6°C and the amplitudes of water temperature increased with river lengths and catchment areas (Scorff R.> Nivelle R.> Oir R.). Time series of mean water and air temperatures appear to be positively aligned, while time series of water amplitudes appeared to be positively aligned with amplitudes of air temperatures but buffered by occurrences of high mean flow (Fig. 6.).

#### Estimates of parameters linking water temperature to air temperature and water discharge

**Figure 7:**
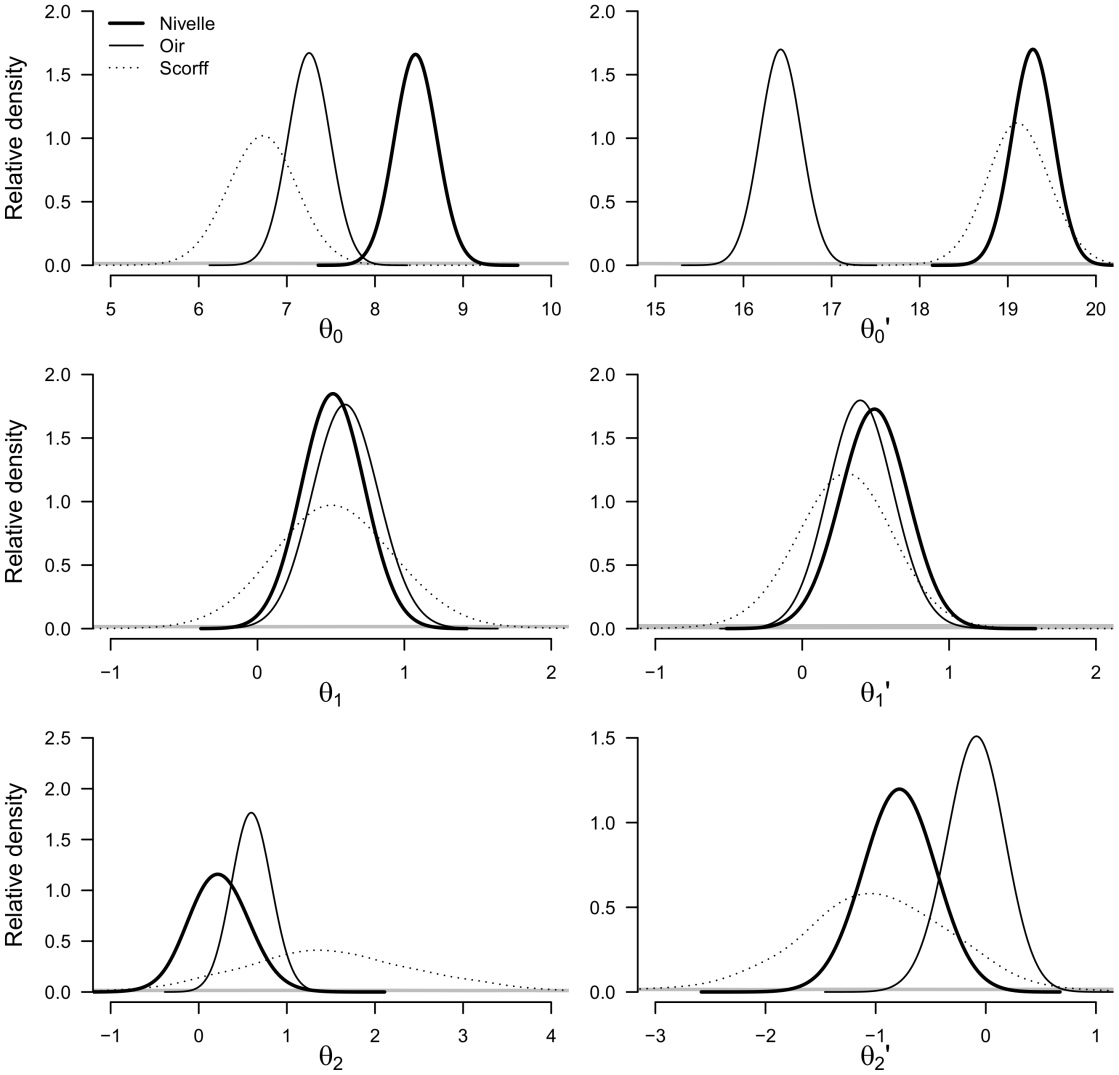
Posterior distributions of the parameters involved in the linear regression used to infer the time series of stream water temperatures based on time series of air temperature and discharge used as predictors.

Posterior estimates of regression parameters linking the characteristics of the water temperature sine signal to that of the air temperature and water discharge are consistent overall with our expectation (Fig. 7). Bayesian posterior distributions of all parameters have only low probability to be negative, except 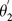. Uncertainties around parameter estimates are greater for the Scorff River, for which data series are shorter (13 years) than those of the Nivelle and Oir Rivers (24 and 22 years respectively). Maximum flow nonetheless had negligible effect on minimum water temperature for the Nivelle River (posterior distribution of 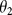 around 0). The relationship between the time series of minimum flow and of the maximum water temperature is also weak (posterior distribution of 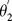 centred around 0) (Fig. 7).

#### Forecasting water temperature from climatic scenarios

**Figure 8:**
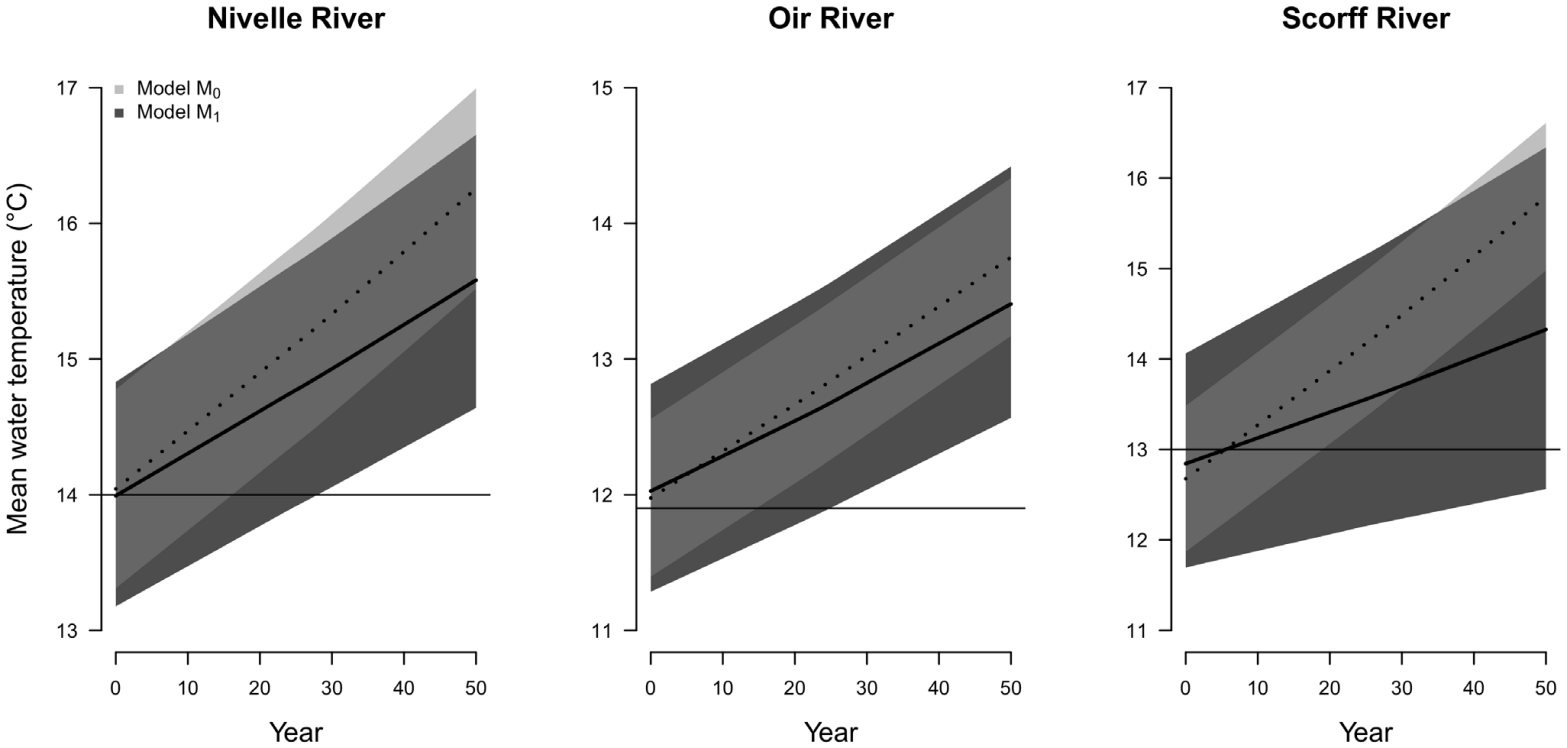
Average temperature calculated for 6 months intervals forecasted by the modelling approaches *M_1_* and *M_0_* on the three rivers over 50 years under an air temperature warming scenario of 3.2°C. Posterior means are represented by solid lines for the model *M_1_* and dotted lines for model *M_0_*. 95% Bayesian credibility intervals correspond respectively to dark and light grey areas for models *M_1_* and *M_0_* respectively. Horizontal Black line: average water temperature observed over the last ten year time series.

Based on the 50 years climatic scenarios, Bayesian forecasts of water temperature produced by both models *M_0_* and *M_1_* exhibit clear increasing trends but the median trends forecast by the modelling approach *M_1_* were significantly weaker (Fig. 8). The mean warming of stream water by the end of the 50 years forecasting period ranges from around 1.5°C to 2.5°C according to model *M_0_* while the three forecast of model *M_1_* were 0.2 to 1.5°C lower.

Meanwhile, the models exhibit strong differences in the uncertainty around forecasts (Fig. 8), the greater predictive performance and quality of fit of model *M_1_* being accompanied by a greater uncertainty in the forecasted trends, with 95% credibility intervals at least 1.4 times greater than from model *M_0_*. The probability that the forecast water temperatures exceeded the average temperature observed in the last ten years can be used as a synthetic metric to compare the forecasts between *M_0_* and *M_1_*. According to model *M_0_*, the number of forecasting years after which this probability exceeds 95% is less than 20 years. According to model *M_1_*, this would occur latter on the three Rivers and are not forecast in case of the Scorff River. Nonetheless, forecasting intervals are still overlapping by the end of the forecasting period (Fig. 8).

#### Reconstruction of missing data using model M_1_

**Figure 9:**
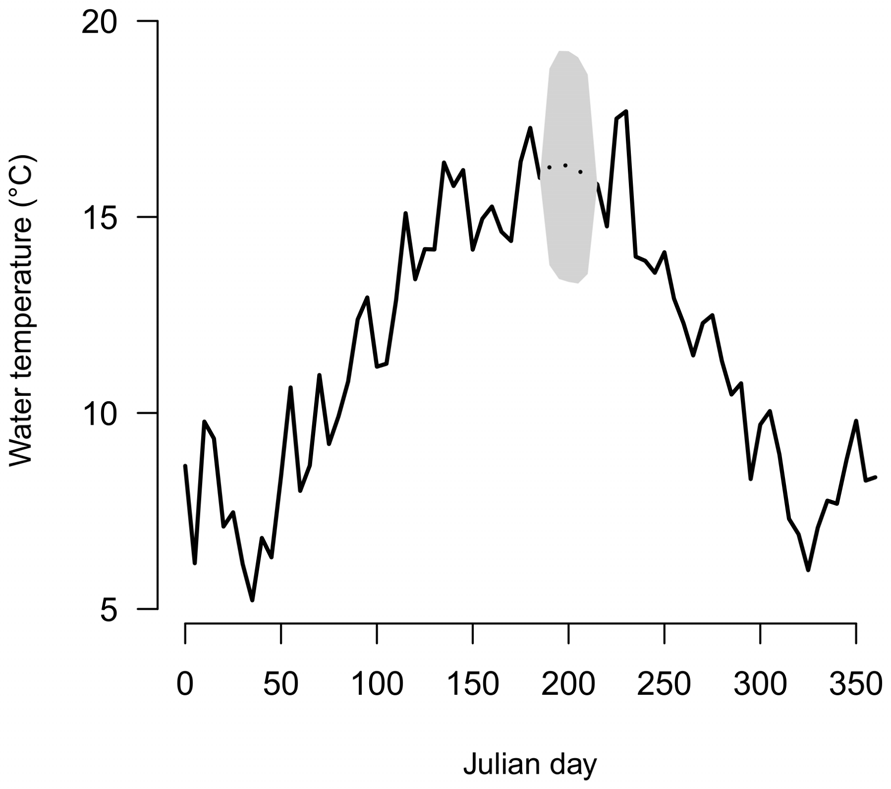
Observed (black line) and estimated missing water temperatures (black dotted line) using model *M_1_* with 95% Bayesian credibility interval (grey area) on the Oir River in 2006.

Missing data are considered in Bayesian modelling as unknown variables that can be estimated directly from their posterior predictive distribution [49]. This is illustrated for the Oir River (Fig. 9) where water temperature reconstructed from model *M_1_* integrate well with the existing time series. Uncertainties around estimates are close to the observed variations of water temperatures around the underlying mean seasonal pattern, showing that estimated water temperatures are good approximation of the true water temperatures.

## Discussion

The effect of climate warming on river temperatures is no longer just speculative, with an observed warming up to 1°C per decade [7], [8], [36]. Providing generic models to reconstruct and forecast water temperature series based on predictors such as air temperature and water discharge, for which historical series or predictions from scenarios of climate change are available, is a prerequisite to better understand how water temperature drives ecosystems functioning, and to evaluate the impact of global warming. In this context, using a statistical framework allowing weighting of the outcomes of different management scenarios is a cornerstone for further biological conservation [48], [59], [60]. The time series modelling approach (*M_1_*) developed in this paper offers a useful contribution to these challenges.

The time series decomposition approach has several advantages over the linear regression modelling (*M_0_*). It can be used to reconstruct continuous time series of discharge, air and water temperatures in the presence of missing data without additional predictors. The method proved useful in reconstructing continuous water temperature time series for three French coastal streams, even with important proportions of the records missing. The time series modelling approach also allows disentangling seasonal periodicity from longer time trends. The illustrative simulated example demonstrated that a simple regression model between air and water temperature does not distinguish between temporal scales lead to different conclusions. Owing to the ability to separate seasonality from long term trends, the methodology based on time series modelling allows for unbiased and more accurate predictions of the water temperature.

When applied to the time series of three coastal streams, the time series modelling approach outperforms the simple linear regression model in quality of fit and predictive performances. The DIC favoured the time series modelling approach despite a higher number of parameters. For the three rivers, cross validation analysis show that the time series modelling approach has better predictive performance than the simple regression. The 50 years forecasting scenarios also shows that the simple regression approach *M_0_* provides unduly warmer forecast of water temperature by comparison with the time series approach *M_1_*. Only the posterior checking, which does not compare the forecasting precision of the two methods, show that the two modelling approaches had equivalent posterior consistency with data.

The time series modelling approach led to greater uncertainty in the forecasts of trends than the simpler linear regression approach. The 95% credibility intervals calculated for the time series modelling approach were at least 1.4 times wider than those of the linear regression model. The lower quality of fit and the lower predictive performances of the linear regression approach indicate that this model is more likely to produce unrealistically precise forecasts. Our approach used only two predictors (air temperature and water discharge) for water temperature. This offers the advantage of a robust approach working from predictors which are easily available from scenarios of climate change. Time series of air temperature and rainfall could be available from downscaling of climatic models [61], and rainfall-runoff models for estimating water discharge from rainfall are widely available [62]. The relevance of including water discharge in our time series modelling approach could be questioned. As pointed out by Koch & Grunewald (2010) [63], rainfall predictions from global and regional climate models are less reliable than air temperature predictions, and the reliability of rainfall–runoff models is also discussed. However, the management of water resource shall be a key issue, for instance in highly irrigated watersheds. Including water discharge in time series modelling is needed to assess the consequences of alternative water resource management choices on water temperature. Sensitivity analyses could be performed using simple scenarios of water discharge evolution and could provide valuable information to managers.

The time series modelling approach could be developed further. A first research direction to improve statistical modelling could consist of implementing more elaborate modelling of random variation around the mean signal. For instance, the covariance between water temperature residuals and the residuals of the predictors could be explicitly incorporated [7], [33]. Observation errors could also be modelled if they are thought to be high and the information to do so is available. Furthermore, additional predictors could also be incorporated. Our time series modelling approach ignores many other factors that may play an important role in controlling water temperature, for example predictable changes in riparian vegetation were not considered in the present study although they have been shown to influence water temperature [64], [65], [66]. The impact of climate change on groundwater discharge and associated spatial heterogeneity of water temperatures, which are of primary importance for the survival of many cold water fish under high temperature stress [67], [68] could be included in the model as well as other climate variables such as evapo-transpiration or solar radiation.

To conclude, the outputs of our model could be used to assess the past and future effects of water temperature on very specific ecological mechanisms such as the growth of juvenile salmonids [12]. More generally, water temperature scenarios are essential inputs for evaluating the effects of climate change on various broader ecological processes such as the dynamics of fish population [15], [69], [70] or food webs [24], [25]. By virtue of the Bayesian framework proposed, river water temperature, an important and yet often unrecorded variable may be estimated and forecasted with its uncertainty fully integrated, an important issue in resource management and ecology.

## Annexe 1

The Oir River flows into the estuarine part of the Sélune River, 8 km from the Bay du Mont Saint Michel. Water temperatures were measured by Tidbit temperature data loggers (±0.2°C) daily at the Cerisel station (Fig. 2) by the National Institute of the Agronomic Research (INRA). Daily air temperatures were obtained from the meteorological station at Saint-Hilaire-du-Harcouët, 14 km east of the water temperature station. Water discharge was measured just upstream of the confluence with the Sélune River, while data were available for 22 years (1986 to 2007), 11.8% of data were missing over the three time series.

The Scorff River flows in to the Atlantic Ocean (Fig. 2). Daily water temperature measurements were made with Tidbit data loggers (±0.2°C) at the Moulin des Princes station by INRA. Daily air temperatures were obtained from the Lorient (Lan Bihoue) airport meteorological station, 9 km south of the water temperature station while discharge was monitored 8 km upstream. Data were available for 13 years (1995 to 2007), 9.6% data were missing for the three series.

The Nivelle River flows from the western Pyrenees, Spain in to the Bay of Biscay at Saint Jean de Luz. Daily water temperature was recorded at Ibarron (Fig. 2) by INRA using successively Jules Richard (±0.4°C), Minilog Vemco (±0.3°C) and Tidbit (±0.2°C) temperature data loggers. Daily air temperatures were recorded 13 km to the north at the Biarritz airport meteorological station. Water discharge was measured at the confluence with its main tributary, the Lurgorrieta. 24 years of data were available (1984 to 2007), with 1% missing across the three time series.
